# Community annotation in biology

**DOI:** 10.1186/1745-6150-5-12

**Published:** 2010-02-18

**Authors:** Raja Mazumder, Darren A Natale, Jessica Anne Ecalnir Julio, Lai-Su Yeh, Cathy H Wu

**Affiliations:** 1 Department of Biochemistry and Molecular & Cellular Biology, Georgetown University Medical Center, Washington, DC 20007, USA; 2 Delaware Biotechnology Institute, University of Delaware, 15 Innovation Way, Suite 205, Newark, DE 19711, USA

## Abstract

Attempts to engage the scientific community to annotate biological data (such as protein/gene function) stored in databases have not been overly successful. There are several hypotheses on why this has not been successful but it is not clear which of these hypotheses are correct. In this study we have surveyed 50 biologists (who have recently published a paper characterizing a gene or protein) to better understand what would make them interested in providing input/contributions to biological databases. Based on our survey two things become clear: a) database managers need to proactively contact biologists to solicit contributions; and b) potential contributors need to be provided with an easy-to-use interface and clear instructions on what to annotate. Other factors such as 'reward' and 'employer/funding agency recognition' previously perceived as motivators was found to be less important. Based on this study we propose community annotation projects should devote resources to direct solicitation for input and streamlining of the processes or interfaces used to collect this input.

**Reviewers:**

This article was reviewed by I. King Jordan, Daniel Haft and Yuriy Gusev

## Introduction

Maximum benefit from genome sequencing efforts can be obtained when as many sequences as possible are associated--or *annotated*--with biological information. Currently, a large part of this biological information (such as the function of a protein) is presented in the scientific literature, but is not captured in any database. Some resources have a limited number of professional annotators who add meaningful information to these sequences based on published experimental results, but the volume of new information precludes the ability to keep pace. One often-proposed mechanism to deal with the problem is to engage the user community in the task.

There are several examples of attempts to engage the community in annotation [1-5] and several articles describe the merits and possibilities of such an approach [6-9]. However, while it is clear that members of the scientific community overwhelmingly use biological databases for research or teaching, they rarely directly contribute (provide annotation) to them.

Here it is necessary to introduce a few terms used within this work, since the phrase "community annotation" can refer to a number of distinctly different activities. *Supervised dispersed community annotation *refers to the case when a coordinator actively contacts experts in the field and requests them to annotate specific items. During a *community annotation jamboree *experts meet (physically or over the internet) and annotate a set of predetermined entries in a given amount of time. Typically this event mixes domain experts and professional annotators. A variant of this is *student community annotation*, which involves annotation by students after they have been taught in class or workshops. Each of the above requires the direct engagement of the community by professional annotators. The version of community annotation requiring the least engagement is *unsupervised dispersed community annotation *(similar to the Wikipedia model), which refers to the case where anyone can log in and annotate an entry of her/his choosing. All of the above forms of community annotation assume that there are expert curators who act as gatekeepers to enforce standards. From a scientific database point of view without such gatekeepers the quality of information will rapidly deteriorate.

It has been observed that both supervised dispersed community annotation and student community annotation are usually successful [10,11]. The most success has come from community annotation jamborees [12,13]. This type of consortium-based annotation jamboree often results in publications and it is possible that this could be the driving force for the success. Alternatively, the amount of direct engagement by professional annotators could be the key to success. Either way, the success is somewhat short-lived since the contributions tend to end with the event. Most unfortunately, what has been considered the Holy Grail of community annotation--sustained contribution via unsupervised dispersed community annotation--has been the least successful.

It is generally believed that the main roadblock to unsupervised dispersed community annotation is based on motivation and incentive issues [14]. It is also possible that there is a simple lack of communication regarding the need for community help. However, there has been no past attempt to determine if these are true. Our experiences with student community annotation led us to question our assumptions about what promotes and what impedes community annotation. We therefore conducted a short survey to better understand interactions between biologists and biological resources, and to hear directly from scientists why there is a lack of contribution. The survey was sent to biologists who have recently published a paper characterizing a gene or protein. The names and e-mail addresses of biologists were obtained by searching PubMed http://www.ncbi.nlm.nih.gov/pubmed/ for articles published in the last five years that describe a protein or gene. Articles were retrieved using a simple keyword search followed by manual inspection of the title and abstract to identify publications which indeed describe experimental characterization of gene/protein function. The corresponding author was then contacted for the survey. The response rate was 33% (50 respondents out of 151 contacted).

## Discussion

Feedback from students involved in an annotation project indicates that coupling training with annotation helped foster better understanding of the tools that were taught in class. Importantly, the feedback led us to consider the factors that might positively influence community annotation: adequate training, clear guidelines as to what constitutes annotation, and a simple annotation interface with clear guidelines for usage. The survey was designed to take into account these considerations in addition to those previously mentioned (incentive and communication).

### Survey

**Figure 1:**
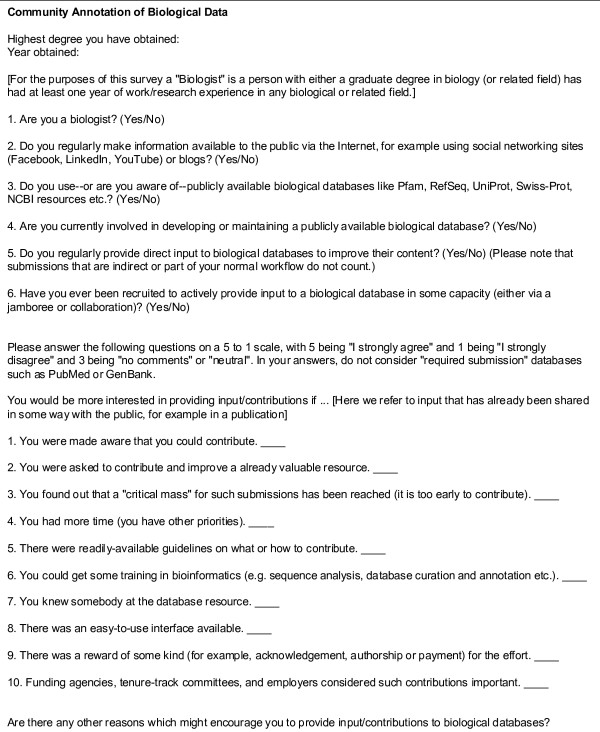
**Survey**.

**Figure 2:**
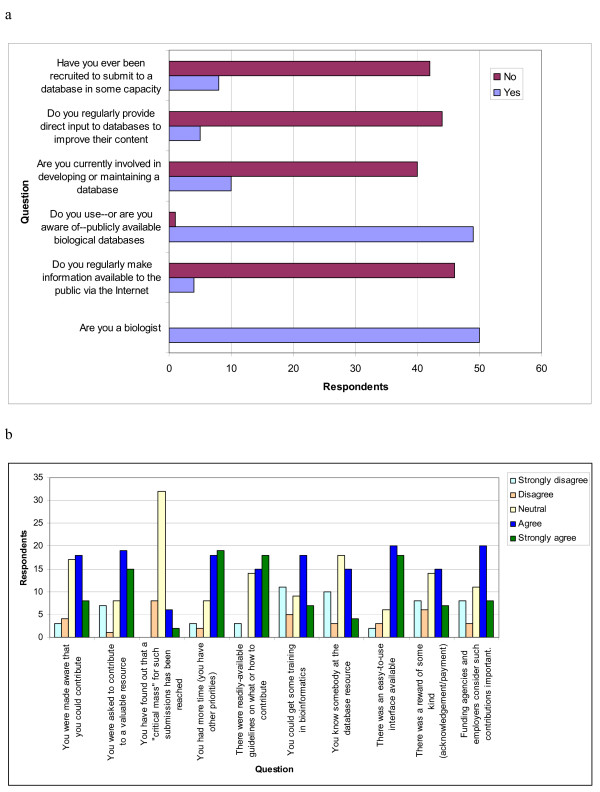
**(a) General survey results (b) Community annotation survey results**.

The survey (Figure 1) consists of two parts. Answers to Part I are Yes/No answers that give an idea of who the participants are. From the results from this section we can see that: a) almost all biologists use publicly available databases; b) very few have been actively recruited to provide annotation to a database; and c) very few of them provide professional or personal information available via the internet (Figure 2a and 2b).

For Part II of the survey people were asked to indicate their position on community annotation using a 5 to 1 scale, (5 being "I strongly agree," 1 being "I strongly disagree," and 3 being "no comments" or "neutral"). Five survey statements had a three-fold difference between the number of people that disagreed (strongly disagree or disagree) vs. agreed (strongly agree or agree) (Supplementary Table 1). These statements are:

You would be more interested in providing input/contributions if...

1. You were made aware that you could contribute (7 disagreed; 26 agreed)

2. You were asked to contribute to a valuable resource (8 disagreed; 34 agreed)

3. You had more time (you have other priorities) (5 disagreed; 37 agreed)

4. There were readily-available guidelines on what or how to contribute (3 disagreed; 33 agreed)

5. There was an easy-to-use interface available (5 disagreed; 38 agreed)

The first two statements are related because if one is asked to contribute to a resource then the person is also made aware that he/she can contribute. The last two statements are also related as an interface is easiest to use when there are clear instructions and readily available guidelines. The third statement gave expected results.

## Conclusions

Based on the results the following become clear:

1. It is obvious that databases need to play an active role in soliciting for annotations.

2. Databases need to invest in developing easy-to-use interfaces and provide simple and clear instructions on what to annotate.

As expected, the survey takers feel strongly that they do not have a lot of time. One could argue that the only way to diminish this impediment is to provide clear instructions and easy-to-use interfaces to save time. Unexpectedly, motivation or credit was not a major consideration, based on the responses.

It is obvious that if community annotation is successful, the future of biocuration would involve not only the professional curators but also the scientific community and the journals [15,16]. We envision that professional biocurators will be needed to perform quality control and standardization.

## Competing interests

The authors declare that they have no competing interests.

## Authors' contributions

RM conceived and designed this study and taught the bioinformatics course. DN participated in the writing of the manuscript, designing the survey and teaching the course. JAJ participated in designing and conducting the survey. LY participated in teaching the course and designing the survey. CW participated in the student annotation part of the project and provided insights on community annotation. All authors read and approved the final manuscript.

## Reviewers' comments

### Reviewer's report 1

I. King Jordan, Georgia Institute of Technology

The authors of this opinion piece weigh in on a critical issue in genomics - the relative paucity of dispersed and/or community efforts at functional annotation. This issue is likely to become ever more pressing as the volume of raw data in biological databases increases without a concomitant increase in actual knowledge. They conducted an interesting survey of 50 biological researchers in order to try and elucidate the root causes of this issue. Their conclusions are slightly unexpected in the sense that rewards or incentives for providing annotations were found to be substantially less important than awareness of the opportunity to annotate and ease-of-use issues with respect to annotation interfaces. This is an interesting article that should prove to be of interest to the readers of Biology Direct. However, the survey employed here was far from scientific, not that the authors necessarily intended it to be, and more to the point there may be some reasons to doubt the main conclusions of the article. I elaborate on these issues below and point to two specific aspects of the work that call into question the perceived lack of importance for a rewards based or incentivized system for annotation. In addition, I raise a couple of specific questions about the survey and approach taken here.

1. Implicit in this manuscript is a very strong endorsement of the role of motivation and incentives in driving community annotation efforts. The authors mention that their own experience with student community annotation led them to question the role of motivation and incentives in driving community annotation. Presumably, their involvement with students was taken as an indicator of the importance of adequate training and clear guidelines for annotation. This would be consistent with the results of the survey and may have also influenced the wording of the questions (see point #2 below). However, one can hardly think of a more motivated or incentivized group of community annotators than students whose classroom performance and grade depend directly on their annotation efforts. Thus, the efficacy of student based community annotation would seem to be an endorsement of both sides of the argument presented here: the need for solicitation, training and guidelines on the one hand as well as the importance of motivation and incentives on the other.

**Authors' response: ***Indeed, we agree with this assessment. Here, we must admit that we previously viewed the notion of successful community annotation of scientific databases with some skepticism. Yet, we found ourselves confronted with what we considered to be a successful outcome with student community annotation. Thus, in actuality, this experience with students did not (directly) lead us to question our assumptions. Rather, it led us to examine the issue of community annotation per se, and we had to consider that our assumptions might be incorrect*.

2. It may be possible that the message of the paper is influenced strongly by the wording of the survey questions. Specifically, I am a bit suspect as to the wording of the two questions related to incentivizing or rewarding community annotation efforts (questions #9 & #10 in part 2 of the survey). The wording of these questions is a bit vague and tepid. For instance, question #9 mentions a 'reward of some kind'. What if the question proposed a specific dollar amount for each gene/protein annotation? Question #10 states that 'Funding agencies ... considered such contributions important.' What if this question was posed as 'You would be significantly more likely to receive a grant.' In both cases, I bet that questions with more tangibly framed rewards would elicit more positive responses. The point is that the framing of the motivations and incentives in the survey may not be strong enough to elicit strong responses. But even with the somewhat vague wording of these questions, substantially more respondents agreed than disagreed with them, which calls into question the notion that these factors are relatively unimportant.

**Authors' response: ***It is true that the questions related to incentive were framed rather generally. We did not believe that we could reasonably put some specific incentive there. For example, if we indicated a dollar amount, what amount to put? What if the amount indicated was too low? We decided instead to allow the taker to imagine the reward. Also, one of the overriding concerns when crafting the survey was that the survey as a whole--and each question--had to be brief. We thus could not explore further, for example, by providing a range of specific dollar amounts--especially if we, as database developers that stand to gain from community annotation, are not in a position to offer such rewards. In any case, it does not appear (to us) that these questions were framed any more or less vague than others*.

3. The importance of question wording in the survey may be even more apparent for question #3 in part 2. After reading this question several times, I still could not understand exactly what was being asked. Apparently, this was true of the survey takers as well since far more people did not have an agree or disagree answer for this question than for any of the other questions.

**Authors' response: ***This was an unfortunate attempt to try to cover any possible reason that came to mind using as brief a phrasing as we could. It was intended to convey the notion of "everyone else is doing it" without sounding too flip*.

4. There is one result I found particularly hard to fathom regarding this survey. It looks like less than 5 out of 50 respondents answered 'Yes' to the rather generically worded question about making public information available on the internet including social networking sites such as Facebook and LinkeIn (see Fig 2a). Is this really possible? Who are these people? Did you search for the survey respondents online to confirm these results? If I were to survey 50 of my colleagues, I don't think I could find a single one who didn't have some information publicly available on the web, be it a lab webpage, a departmental webpage or their LinkedIn or Facebook sites. I'm just curious about this result.

**Authors' response: ***The purpose of this question would have been fulfilled if the responses indicated something along the lines of "there's nothing anyone could do to make me engage in community annotation." In such case, the responses here would help discern if there was just some general problem with doing anything at all online (indeed, such nuances could be explored using the survey results; we reported only the most obvious conclusions). It is entirely possible that the respondents do have such web pages. However, the wording of the question--"regularly" and "make information available"--might have come into play here. If one were to interpret that question as we intended, a positive response would be elicited only if the taker provided information that was not generally available to the public already. Indeed, one of the authors only recently signed on to sites such as those mentioned by the reviewer, and even then does not make any information available that cannot already be found elsewhere. It is also possible that the responses are a function of the age of the respondents. A cursory look at estimates of the average age of internet users indicates that it falls somewhere between 30-40 years. Since we contacted the (presumed) principle investigator, it is not too unreasonable to think such websites are not part of the everyday routine for many respondents*.

5. I was confused as to one point. Why is unsupervised dispersed community annotation the 'Holy grail' of community annotation given the importance that the authors place on quality control by expert curators or 'gatekeepers'?

**Authors' response: ***This mechanism would, presumably, require the least amount of curator time, thus freeing up manpower to tackle looming backlogs. Nonetheless, curating a database requires quite a bit of training, especially with respect to standards such as controlled vocabularies, and having a gatekeeper thus trained would provide a way to maintain these standards while still harnessing the knowledge of the community. The term "unsupervised" as defined in the paper refers to the situation where annotation is being provided without prior direct contact by database providers, and does not indicate a lack of downstream processing of the information provided*.

6. Having raised the issues with the manuscript that are enumerated above, I would like to point out that one can hardly argue with the two conclusions that 1) databases need to more actively solicit annotations and 2) databases need easy to use annotation interfaces. However, in general I would like the authors to more carefully consider the role, or lack thereof, for motivations and incentives in the process as they relate to their findings.

**Authors' response: ***We wholeheartedly agree. It was not our intention to minimize the role that incentives might play, and we were actually surprised at the findings. Certainly further probing into the matter is warranted*.

### Reviewer's report 2

Daniel Haft, The J. Craig Venter Institute

The scientific community thrives on the free, open, and efficient spread of information. Yet clearly something is broken about information's flow from the laboratory where it is generated to the large, searchable public databases that help enable research. It hurts all of us that so much critical information about genes and proteins remains hidden in the primary literature, undigested and unavailable. Community annotation would be a good solution if barriers to achieving it could be overcome. It looks like we face "the tragedy of the commons" (PMID:9563937); we would each be better off if everybody made the requisite contributions, but nobody individually can realize much benefit from his own good behavior. But is incentive and reward really the reason community annotation has achieved only limited results so far? Or are other technical barriers of greater importance?

Mazumder, et al., part of the American branch of the team that produces UniProt, surveyed a number of experimentalists to investigate what are the real barriers to community annotation, in case they were not as we thought. Their results may be skewed somewhat towards optimism, coming from the self-selected group of those who went so far as to allocate some time to answer the survey. A response rate for the survey would be informative. But these results contain some real surprises. Rather than a generalized time crunch and a lack of clear reward, the shortage of community annotation may simply reflect that we failed to ask the community boldly and clearly for their help, and failed to make contributing as easy as it needs to be. If better methodology is all that is needed, then that is a call to action. There are, after all, examples of pretty good (if imperfect) success from unpaid contributions of intellectual content for the common good. These include both Wikipedia and the scientific peer review system.

**Authors' response: ***The response rate was 33% (50 respondents out of 151 contacted). This information has been added to the paper*.

### Reviewer's report 3

Yuriy Gusev, Georgetown University

This is a comment/discussion notes type of paper.

This manuscript addresses an important issue of gene and protein function annotation.

This is important and timely commentary as in my opinion the gargantuan task of biology annotation is currently lying on the shoulders of small cohort of professional annotators. This process is therefore is rather slow and lagging significantly behind of publications on gene function.

However the importance of functional annotation cannot be underestimated as it is critically important and vital part of modern biology in general and of any quantitative biology efforts in particular. The paper is aimed at initiating a dialog and a discussion within the biological community on this important topic and I wholeheartedly support this idea.

I have no objections for publishing this manuscript
